# An atomistic study of sticking, bouncing, and aggregate destruction in collisions of grains with small aggregates

**DOI:** 10.1038/s41598-024-57844-y

**Published:** 2024-03-28

**Authors:** Maureen L. Nietiadi, Herbert M. Urbassek, Yudi Rosandi

**Affiliations:** 1 Department of Geophysics, Universitas Padjadjaran, Jatinangor, Sumedang, 45363 Indonesia; 2 Physics Department, University Kaiserslautern-Landau, Erwin-Schrödinger-Straße, 67663 Kaiserslautern, Germany

**Keywords:** Collisions, Molecular dynamics, Dust, Ice, Astronomy and astrophysics, Space physics, Planetary science, Materials science

## Abstract

Molecular dynamics simulations are used to study central collisions between spherical grains and between grains and small grain aggregates (up to 5 grains). For a model material (Lennard-Jones), grain–grain collisions are sticking when the relative velocity *v* is smaller than the so-called bouncing velocity and bouncing for higher velocities. We find a similar behavior for grain–aggregate collisions. The value of the bouncing velocity depends only negligibly on the aggregate size. However, it is by 35% larger than the separation velocity needed to break a contact; this is explained by energy dissipation processes during the collision. The separation velocity follows the predictions of the macroscopic Johnson–Kendall–Roberts theory of contacts. At even higher collision velocities, the aggregate is destroyed, first by the loss of a monomer grain and then by total disruption. In contrast to theoretical considerations, we do not find a proportionality of the collision energy needed for destruction and the number of bonds to be broken. Our study thus sheds novel light on the foundations of granular mechanics, namely the energy needed to separate two grains, the difference between grain–grain and grain–aggregate collisions, and the energy needed for aggregate destruction.

## Introduction

Collisions between nanoparticles are relevant in several scientific and technological contexts ranging from chemical engineering to geology^1^, such as in aerosol physics^2^ or in adhesive particle flows^3^. They have been studied with particular emphasis in a space environment, where collisions between dust particles are ubiquitous; they occur in protoplanetary dust disks^4,5^ but also in evolved planetary systems where dust may originate from comets^6,7^ or asteroid collisions^8–10^. They are also found in planetary rings^11^, planetary nebulae^12^ and even in cold starless and prestellar cloud cores^13,14^. Collisions between dust particles may lead to dust coagulation or dust shattering depending on the size of the collision partners, their collision velocity and other factors and thus decide on the fate of the dust population^15^; in the context of protoplanetary disks, such collisions govern the early stages of planet formation^16^.

Often, granular mechanics codes are used to describe collisions between dust aggregates^17–19^. However, such codes use macroscopic concepts of contact mechanics – such as the break-up energy^17,18,20^ – whose validity for nanoparticles is not always clear. Molecular dynamics simulations of selected collision events may help to elucidate the contact mechanics of nanoparticles. Such simulations were successfully performed in fields ranging from nanotribology^21,22^ to nanoscale plasticity^23^.

Also the collision mechanics of nanoparticles has been investigated using molecular dynamics simulation^24,25^, but up to now only the collision between two individual grains has been studied. A prime quantity of interest is the so-called bouncing velocity which describes the threshold between low-velocity sticking and high-velocity reflecting collisions; its value strongly influences grain coagulation and hence the post-collisional size distribution of the dust population.

In the present paper, we address the question of how the bouncing process changes if individual grains collide with a grain aggregate rather than with other isolated grains. We use a simple model for interatomic interaction, the Lennard-Jones potential, since it may be considered as a prototypical material where the results obtained obey simple scaling rules that often allow their transfer to other systems of interest^26–28^. Also, in view of the computational costs of molecular dynamics simulations, the aggregates studied are restricted to small and highly symmetric cases. Our results will allow to assess the predictions of granular mechanics on the bouncing from and the destruction of aggregates by collisions with individual grains.

Our study thus focuses on one of the foundations of granular mechanics, namely the energy needed to separate two grains (that is the so-called separation or breakup energy^20^) and the difference in collisions between two grains and between a grain and a (small) aggregate. This study is thus part of a multiscale approach that attempts to base granular mechanics on atomistics.

## Method

Atoms interact via the standard Lennard-Jones (LJ) potential

1 $$\begin{aligned} V(r) = 4 \epsilon \left[ \left( \frac{\sigma }{r} \right) ^{12} - \left( \frac{\sigma }{r} \right) ^{6} \right] \end{aligned}$$

with length parameter $\sigma$ and energy parameter $\epsilon$; the potential is cut off at $r_c=2.5\sigma$ such that $V(r)=0$ for $r>r_c$. In the following, we will use LJ units: Lengths are measured in units of $\sigma$, energies in units of $\epsilon$, and masses in units of the atomic mass *m*. The unit of time is $\tau =\sigma \sqrt{m/\epsilon }$ and the unit of velocity is $\sqrt{\epsilon /m}$.

Amorphous LJ grains are generated using the recipe of Ref.^29^, which is based on rapidly quenching a molten volume^30,31^. A sphere of radius *R* containing *N* atoms is cut out from the amorphous material and then relaxed in an NVE ensemble for a LJ time of 25 in order to obtain relaxed surfaces. The final temperature of the material is around 0.025. We use grains with a radius of $R=80$, such that a grain contains $N= 2\,196\,273$ atoms and has a mass of $M= 2\,196\,273$.

**Figure 1:**
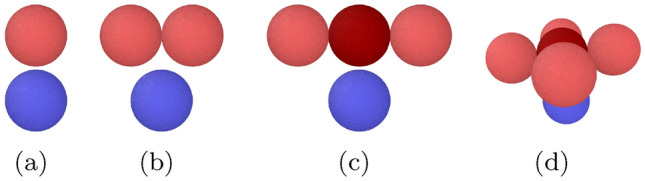
Collision scenarios: a projectile grain (blue) collides with a (**a**) monomer, (**b**) dimer, (**c**) trimer, and (**d**) cross-shaped planar pentamer. The grains of the target aggregates are colored red and the central monomers in the trimer and pentamer are colored dark red. The projectile impinges centrally on the center of mass of the aggregate; the relative collision velocity is perpendicular to the aggregate axis (b, c) or plane (d).

Besides shooting a grain against another grain, Fig. 1a, we also consider shooting a grain against a small aggregate of grains. Only simple symmetrical aggregates are considered, namely a dimer, Fig. 1b, a trimer, Fig. 1c, and a cross-shaped planar pentamer, Fig. 1d. For easier notation, the collision of the grain with another grain will be denoted as the collision with a monomer. The aggregates are built from the individual grains by placing the grain centers such that the grains are in the attractive part of the interaction potential; the aggregate is then relaxed carefully to obtain well relaxed necks between the grains.

**Figure 2:**
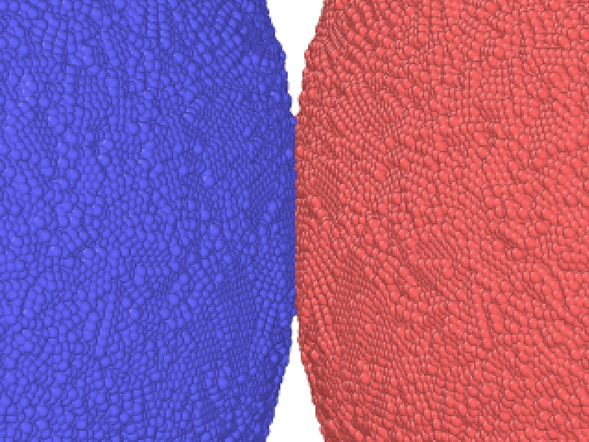
Neck forming between two neighboring grains. Atoms are colored according to their grain affiliation.

According to the macroscopic Johnson–Kendall–Roberts (JKR) theory, the equilibrium contact radius of two grains amounts to^32,33^,

2 $$\begin{aligned} a_{\textrm{JKR}} = \left( \frac{9\pi R^2 \gamma }{2 E_{\textrm{ind}}} \right) ^{1/3}, \end{aligned}$$

with the surface energy $\gamma$ and the indentation modulus $E_{\textrm{ind}}$. Using the material values for the amorphous LJ grains given in Sect. 3.1 below, this amounts to $a_c= 6.47$. Our simulation results give $a_c= 6.52$ in good agreement with the JKR prediction. Fig. 2 gives an atomistic view of the intergranular neck forming between two adjacent grains.

In this study, we only consider central collisions, in which the projectile grain is directed versus the center of mass of the target aggregate. All collisions are performed in the center-of-mass system, and also the evaluation of kinetic energies only refers to the center-of-mass system. The relative velocity, ${\varvec{v}}$, is perpendicular to the aggregate axis (for dimer and trimer collisions), and perpendicular to the aggregate plane for the collision with the pentamer.

We found that the amorphous grains are sticking for central impacts for all velocities; this is in contrast to crystalline LJ grains^34,35^. In order to improve the tendency for bouncing, we modified the attraction between atoms of different grains by reducing the LJ parameter $\epsilon$ in Eq. (1) to a parameter $\epsilon _{12}$, while the interaction between atoms of the same grain is unaltered. Since collisions of amorphous LJ grains with $\epsilon _{12} \ge 0.25$ are sticking for all velocities^29^ we choose $\epsilon _{12}= 0.1$.

We note that also in previous simulation studies of LJ grain collisions, a reduction of the intergranular attraction was introduced^29,36–40^.

The simulations are run until the 2 collision partners separated from each other at least by the cut-off radius of the potential. If the grains stick after the collision, the termination of the simulation is to some degree arbitrary; for determining the lower bouncing velocity, $v_{\textrm{bounce}}$, we waited a factor of 5–10 longer than the nearest bouncing case to see if the 2 grains are really stuck together.

The molecular dynamics simulations are performed with the LAMMPS code^41^. Atomistic snapshots are generated with OVITO^42^.

## Results

We shall discuss first the so-called separation velocity and energy, since these concepts are often used to provide a scale for collision energies in granular mechanics; they provide the appropriate background to discuss grain bouncing and aggregate destruction. After presenting the results for central grain–grain collisions, the collisions of a grain with the central grain of a trimer and pentamer will be evaluated. Finally, grain–dimer collisions will be investigated, in which the projectile grain hits the dimer centrally and therefore the dimer grains obliquely; this collision geometry strongly changes the collision dynamics with respect to that of the trimer and pentamer collisions.

### Separation velocity

**Figure 3:**
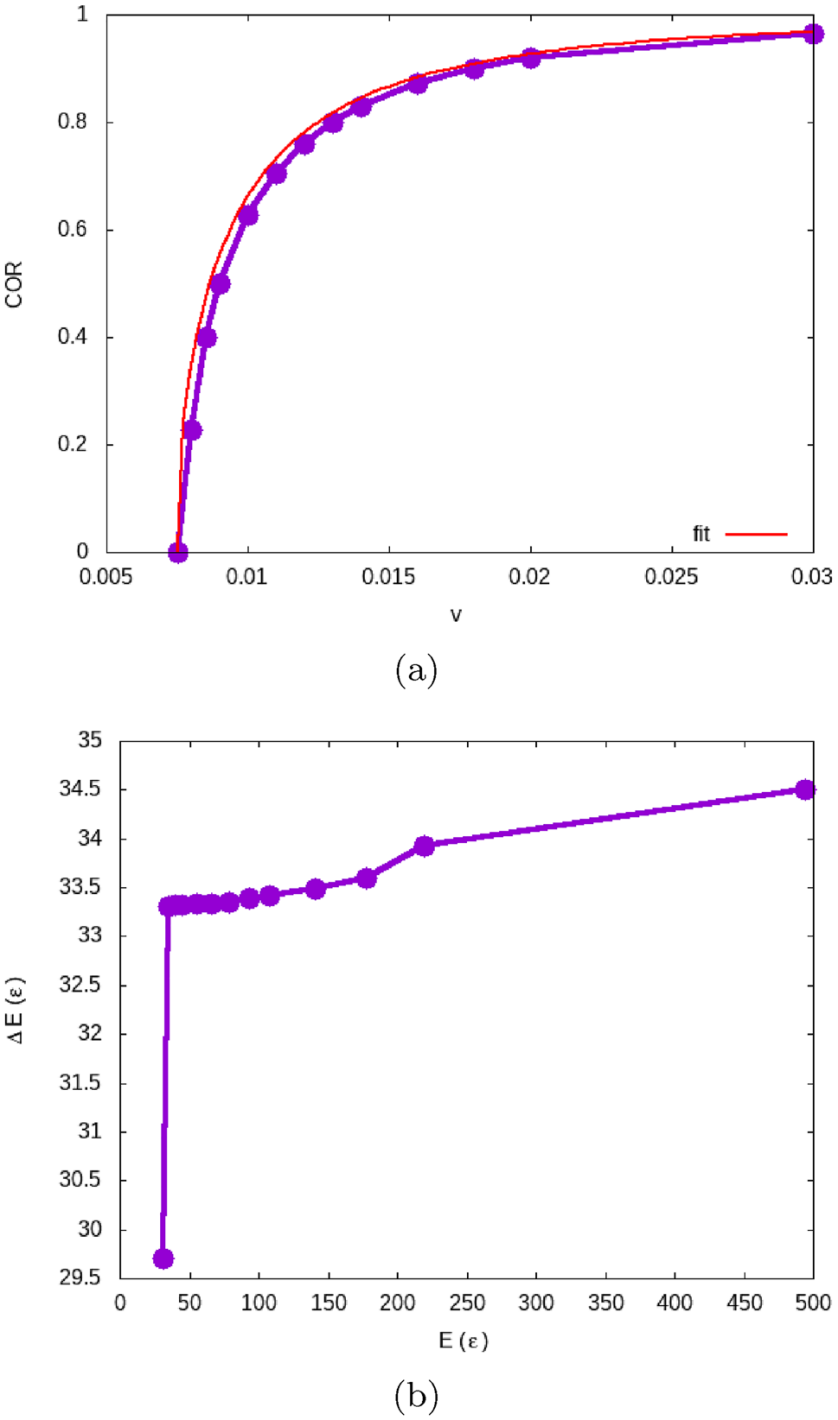
Separation process of a grain–grain dimer: (**a**) shows the velocity dependence of the COR, and (**b**) the energy dependence of the energy loss. The curve in (a) is a fit to Eq. (3) using $v_{\textrm{sep}}=7.75\cdot 10^{-3}$.

The energy needed to break a grain–grain contact, $E_{\textrm{sep}}$, plays an important role in granular mechanics simulations of aggregate collisions^18,20^. Also denoted as the breakup energy, it is used to determine whether an aggregate collision leads to grain losses or even total aggregate destruction^20,43^. We determine it by considering a relaxed dimer consisting of grains 1 and 2; we give each atom in grain 1 a velocity $+v/2$ along the axis joining the two grain centers in the direction opposite from the neighboring grain and each atom in grains 2 a velocity $-v/2$. Thus initially, the grains attempt to separate with relative velocity *v*. We denote as the separation velocity, $v_{\textrm{sep}}$, the minimum velocity *v* needed to actually separate the two grains; the separation energy is then given by $E_{\textrm{sep}}= (M/4)v_{\textrm{sep}}^2$.

We denote the ratio of the velocity $v'$ of a grain after separation to the initial velocity as the coefficient of restitution (COR); values of COR > 0 denote grain separation. Fig. 3a shows the COR for this scenario. The highest velocity where COR = 0 is $7.5\cdot 10^{-3}$ and the smallest velocity where COR > 0 (and hence the grains separate) is $8\cdot 10^{-3}$; we thus determine $v_{\textrm{sep}}=7.75\cdot 10^{-3}$ and $E_{\textrm{sep}}= 33.0$. Fig. 3b displays the energy loss, $\Delta E = E'-E$, during the separation process; in the velocity range considered, the energy loss stays close to the value $E_{\textrm{sep}}$ and only increases slightly with collision energy.

The velocity dependence of the COR in Fig. 3 can be modeled by a law^44,45^

3 $$\begin{aligned} \textrm{COR}(v) = \sqrt{1- \left( \frac{v_{\textrm{sep}}}{v} \right) ^2} . \end{aligned}$$

It originates from the idea that the collision partners suffer a constant energy loss $W= (M/4)v_{\textrm{sep}}^2$ during the collision. Then the kinetic energy after the collision amounts to $E' = E-W$, and since $E=(M/4)v^2$, and $E'=(M/4)v'^2$ with the post-collision relative velocity $v'$, we have

4 $$\begin{aligned} \frac{1}{4} Mv'^2 = \frac{1}{4} Mv^2 - \frac{1}{4} M v_{\textrm{sep}}^2 , \end{aligned}$$

from which immediately derives Eq. (3). As Fig. 3 demonstrates, the separation process well fulfills the hypotheses underlying the law, Eq. (3); the corresponding curve has been added to Fig. 3a and well fits to the data.

The macroscopic JKR theory predicts the separation velocity to be^44–46^

5 $$\begin{aligned} v_{\textrm{sep}}= \left( \frac{C}{\rho } \right) ^{1/2} \left( \frac{\gamma ^5}{E_{\textrm{ind}}^2 R^5} \right) ^{1/6} . \end{aligned}$$

Besides the grain radius *R*, the surface energy $\gamma$, the indentation modulus $E_{\textrm{ind}}$, and mass density $\rho$ determine the bouncing velocity. Here, $E_{\textrm{ind}}= Y/(1-\nu ^2)$ is determined from the Young’s modulus *Y* and the Poisson ratio $\nu$ of the material. *C* is a constant which – depending on the model assumptions – assumes values between 0.30 and 18.3^20,44–46^. The value of *C* depends on the energy dissipation processes that the macroscopic calculations include during the separation process such as the excitation of elastic waves, and also viscoelastic or plastic processes in the separating grains. We note that molecular dynamics simulations such as they are performed in the present study are more adequate to describe inelastic processes (in particular, plasticity) during the collision than macroscopic continuum models.

For an amorphous LJ material, we use^40^ $E_{\textrm{ind}}= 53.5$ and $\rho =1.0$. The value of the surface energy is proportional to the intergranular energy $\epsilon _{12}$ such that^40^

6 $$\begin{aligned} \gamma = 1.63\epsilon _{12} , \end{aligned}$$

and Eq. (5) simplifies to

7 $$\begin{aligned} v_{\textrm{JKR}} = 0.40 C^{1/2} \left( \frac{\epsilon _{12}}{R} \right) ^{5/6} . \end{aligned}$$

We note that this result is almost identical, for $\epsilon _{12}=1$, with that for crystalline LJ grains^47^.

For $R=80$ and $\epsilon _{12}=0.1$, Eq. (7) thus predicts

8 $$\begin{aligned} v_{\textrm{JKR}} = 1.52 \cdot 10^{-3} \cdot C^{1/2} . \end{aligned}$$

Our simulation result of $v_{\textrm{sep}}=7.75 \cdot 10^{-3}$ is well described by the continuum expression, Eq. (8) with $C=26.0$, at the upper end of the range of the predicted values^20,44–46^. The high value is caused by the energy dissipation processes during the breaking of a well relaxed contact.

### Grain–monomer collisions

**Figure 4:**
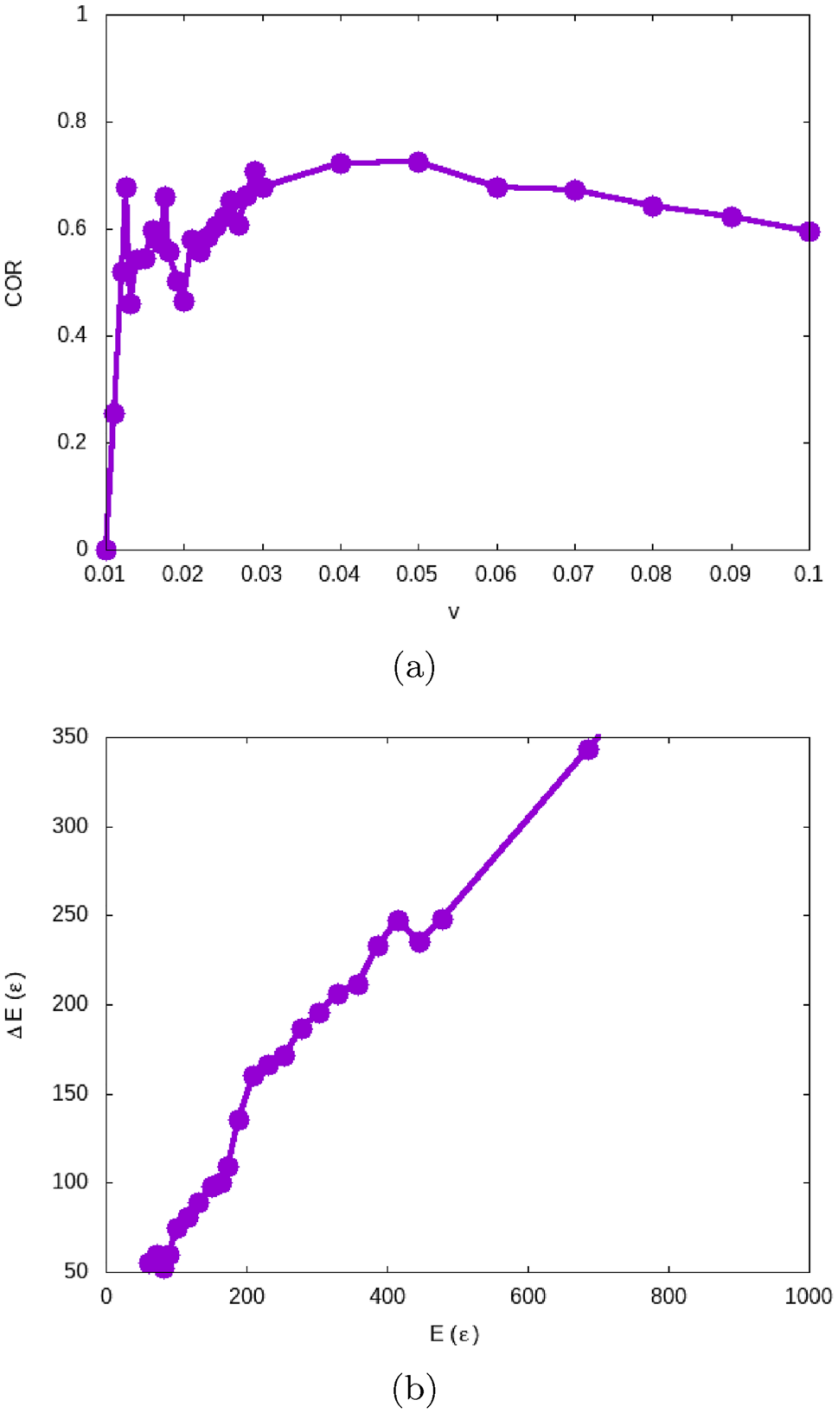
Dependence of (**a**) the coefficient of restitution (COR) on velocity and (**b**) the energy loss on collision energy for grain–grain collisions.

**Table 1:** Bouncing energy, $E_{\textrm{bounce}}$, and bouncing velocity $v_{\textrm{bounce}}$, taken as the average, Eq. (10), of $v^<$ and $v^>$, which denote the largest velocity at which the grains stick and the lowest velocity at which grains bounce, respectively.

Target	$v^<$ ($10^{-3}$)	$v^>$ ($10^{-3}$)	$v_{\textrm{bounce}}$ ($10^{-3}$)	$E_{\textrm{bounce}}$
monomer	10	11	10.5	60
dimer	9	12	10.5	81
trimer	11	12	11.5	109
pentamer	9	12	10.5	101

Collisions between two grains will also be denoted as grain–monomer collisions. Fig. 4a displays the dependence of the COR on impact velocity *v*. For grain–monomer collisions, the COR is defined as the ratio of the projectile relative velocity after the collision, $v'$, to its initial velocity, *v*:

9 $$\begin{aligned} \textrm{COR}= | v'| / v . \end{aligned}$$

The collision with a monomer constitutes the paradigmatic case of a central collision of two grains which has repeatedly been studied for various materials with atomistic simulation^24,25,48^. The COR features a low-velocity sticking regime (COR = 0) and a high-velocity bouncing regime (COR > 0); the bouncing velocity, $v_{\textrm{bounce}}$, separates these two regimes. The largest sticking velocity, $v^<$, and the smallest bouncing velocity, $v^>$, bracket $v_{\textrm{bounce}}$, such that we use the arithmetic mean as the bouncing velocity,

10 $$\begin{aligned} v_{\textrm{bounce}}= \frac{v^< + v^>}{2} . \end{aligned}$$

Our simulation result, $v_{\textrm{bounce}}= 10.5 \cdot 10^{-3}$, Table 1, can be compared to the separation velocity $v_{\textrm{sep}}= 7.75 \cdot 10^{-3}$, which describes the velocity needed to destroy an established grain–grain contact, see above: $v_{\textrm{bounce}}\cong 1.4 v_{\textrm{sep}}$. Accordingly, the corresponding energies differ by a factor of around 2. This demonstrates that energy dissipation processes during the collision influence the collision dynamics and exceed the dissipation during breaking a contact.

Our present result can be compared with the previous result^29^ on the bouncing of grains with $R=88.23$ and $\epsilon _{12}=0.2$ which gave a bouncing velocity of $v_{\textrm{bounce}}= (17 \pm 3) \cdot 10^{-3}$; the two results compare well when scaling *R* and $\epsilon _{12}$ according to Eq. (7). That previous study^29^ also showed that the value of $v_{\textrm{bounce}}$ is subject to an uncertainty of around 20 %; which is caused by the surface roughness of amorphous grains. We note in addition that for the higher value of $\epsilon _{12}=0.2$ used in that study, grain bouncing ceased for higher velocities, $v \sim 0.3$, such that a bouncing window exists similar to that in the collision of crystalline LJ grains^35^. For the present case of $\epsilon _{12}=0.1$, bouncing persists up to the highest velocity simulated, $v=1$, see the data in the Supplementary Material (SM).

The velocity dependence of the COR in Fig. 4a initially shows a steep increase with velocity, followed by a slow decrease. The velocity increase roughly follows the square-root dependence expected for a constant energy loss during the collision, see Eq. (3). Towards even higher velocities, the COR decreases since energy dissipation processes – in particular plastic processes during the collision – increase the energy loss, such that the assumption of a constant energy loss, basic to Eq. (3), fails. The grain deformation at higher collision velocities has been amply demonstrated for the example of two-grain collisions in previous work^29^.

The large COR fluctuations seen in the velocity region above the bouncing threshold, $v=0.01$–0.022, are caused by the surface roughness and disordered structure of the amorphous grains, compare Fig. 2. As for larger velocities the contact area encompasses larger surface regions of the colliding grains, these individual structures are reflected in the energy loss during collisions and hence in the COR.

Fig. 4b plots the energy loss as a function of the collision energy. The bouncing velocity corresponds to a collision energy of $E_{\textrm{bounce}}= (M/4) v_{\textrm{bounce}}^2= 60$. The energy loss, $\Delta E$ can be calculated from the COR, $v'/v$, simply by $\Delta E=E [1-(v'/v)^2]$. At the bouncing threshold, it is $\Delta E =E$, as it should; a zoom of the function $\Delta E(E)$ in the low-energy region is provided in the SM for closer inspection. Fig. 4b emphasizes that the energy loss during the collision is not independent of the collision energy but increases roughly in proportion to it. This is in contrast to the separation process, in which the energy loss only increased by around 3 % for energies up to $E=500$, see Fig. 3b. This feature is important as it shows that the collisional energy loss is not a materials property but depends also on the collision dynamics itself; in particular, it is considerably larger than the separation energy except at the bouncing threshold. At higher collision energies, $E=700$, corresponding to velocities of $36 \cdot 10^{-3}$, the energy loss amounts to about half the collision energy itself.

As noted above, the energy needed to break a contact is a fundamental quantity in granular mechanics calculations^18,20,43^. As the difference between the energies $E_{\textrm{sep}}$ and $E_{\textrm{bounce}}$ demonstrates, this quantity is not easily defined, since it depends on the dynamics of the contact breaking process – destruction of an existing contact on the one hand, or a collision process in which a contact first is generated and then destroyed on the other hand – and also on the velocity with which it proceeds, see Fig. 4b and Fig. 3b. This difference between the dynamic and static breaking of grain–grain contacts has been discussed previously in the context of macroscopic models, where additional parameters such as a viscous time scale are introduced to characterize it^45^, which are often poorly known.

**Table 2:** Similar to Table 1 but for the (total) destruction velocity, $v_{\textrm{destr}}$, and the destruction energy, $E_{\textrm{destr}}$. $N_c$ gives the number of contacts of the hit grain which is identical to the total number of contacts in the aggregate.

Target	$v^<$ ($10^{-3}$)	$v^>$ ($10^{-3}$)	$v_{\textrm{destr}}$ ($10^{-3}$)	$N_c$	$E_{\textrm{destr}}$
Monomer	–	–	–	–	–
Dimer	15	20	17.5	1	224
Trimer	40	50	45	2	1 668
Pentamer	45	54	49.5	4	2 242

### Grain–trimer and grain–pentamer collisions

The central collision of a grain with a linear trimer, Fig. 1c, is similar to the collision of the projectile with a single grain; however, this grain is now bonded to two neighbors. Also, the collision with a cross-like planar pentamer, Fig. 1d, gives a comparable scenario, but now the hit central grain is four-fold coordinated. We thus expect that these simulations also provide insight into grain collisions with (longer) chains of grains, where one target grain is hit centrally. We note that the collision of the projectile with a monomer, a trimer and a pentamer differ mainly in the number of contacts $N_c$ of the hit central grain; in our simple scenario, $N_c$ is also the total number of contacts in the aggregate.

The collision energy *E* varies in these collision scenarios even for constant relative velocity *v*. For an aggregate consisting of *n* grains, the relative mass of the projectile-aggregate system is $\mu = [n/(n+1)] M$, and hence

11 $$\begin{aligned} E = \frac{1}{2} \mu v^2 = \frac{1}{2} \frac{n}{n+1} M v^2 = \frac{2n}{n+1} E_1 , \end{aligned}$$

where $E_1=(M/4)v^2$ is the collision energy in a grain–grain collision. At identical *v*, the collision energy of grain–aggregate collisions is therefore higher than for grain–grain collisions and can reach twice that value for large aggregates *n*.

Since we work in the center-of-mass frame, the velocity change of the projectile grain – and hence the COR, Eq. (9) – can still be used to assess projectile bouncing. At low velocities, where $v'=0$, COR = 0 implies that also the aggregate has vanishing velocity, because of momentum conservation, and thus denotes a sticking collision. At higher velocities, the projectile bounces from the aggregate.

**Figure 5:**
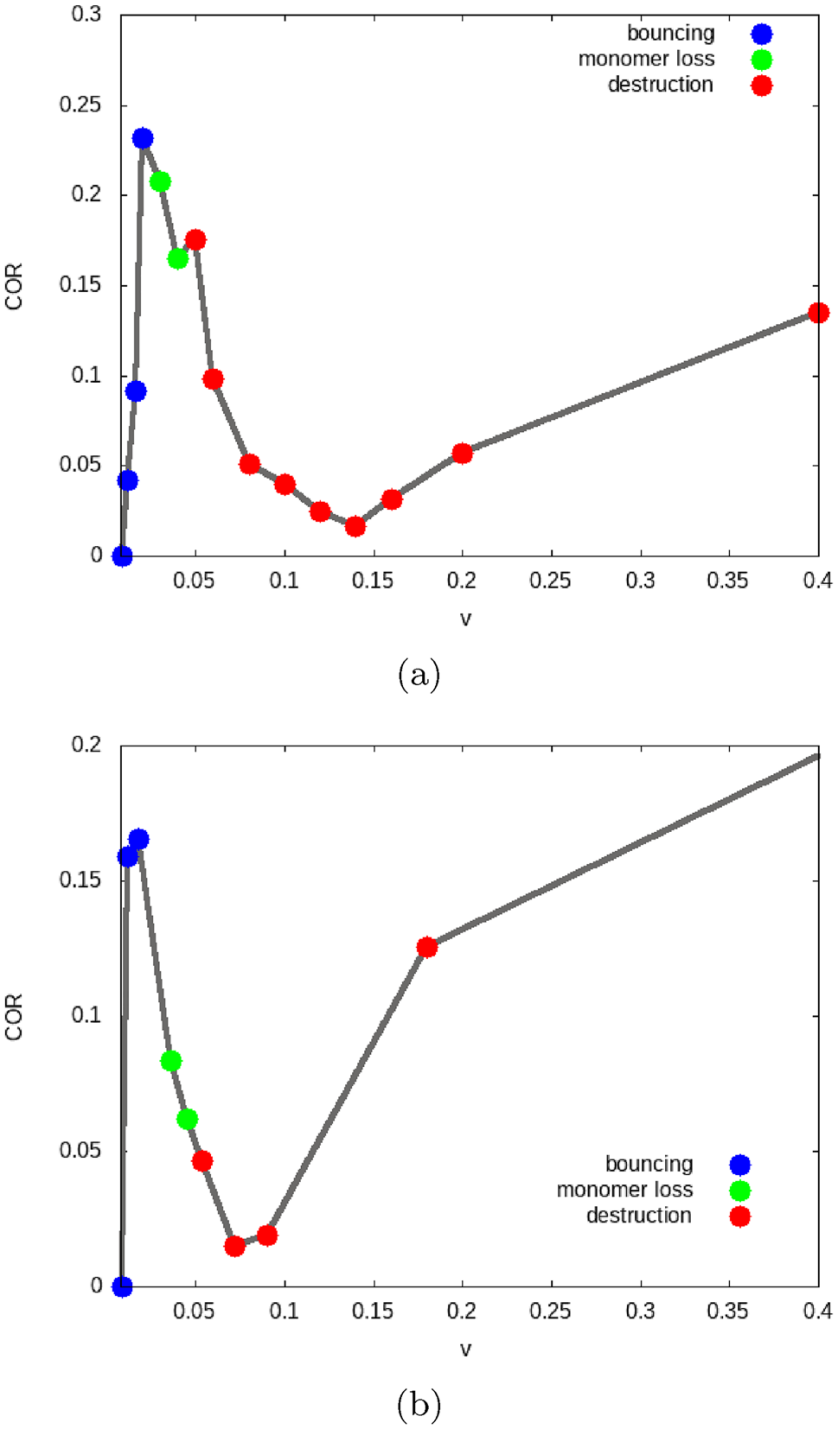
Velocity dependence of the coefficient of restitution (COR) for grains colliding centrally with (**a**) a trimer, (**b**) a pentamer. Data are colored according to the collision outcome: bouncing from the intact aggregate (blue), loss of one monomer inducing aggregate restructuring (green), total destruction (red).

Figure 5 plots the COR and allows us to determine the bouncing velocities $v_{\textrm{bounce}}$. COR data for a wider range of velocities are provided in the SM. The values of $v_{\textrm{bounce}}$ are tabulated in Table 1 which shows that the bouncing velocities are in all cases of similar magnitude. In the central collisions studied here, the projectile grain interacts only with the central grain of the aggregate; this fact explains the similar bouncing velocities.

The bouncing energies, Table 1, show a slight increase with aggregate size *n*, due to the increase of *E* with *n* even for similar values of $v_{\textrm{bounce}}$, see Eq. (11).

**Figure 6:**
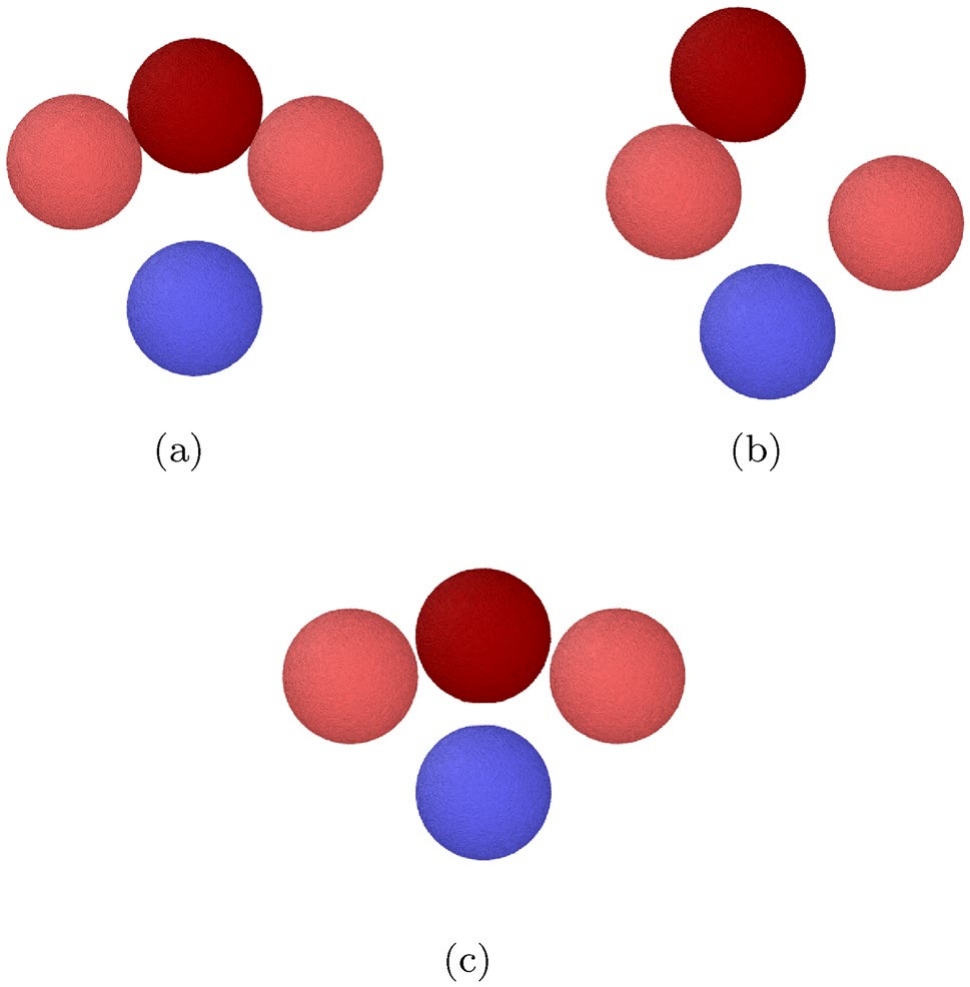
Snapshots showing the collision outcomes for a trimer collision. (**a**) $v= 0.02$ (bouncing). (**b**) $v= 0.04$ (partial destruction). (**c**) $v= 0.1$ (complete destruction). All grains lie in one plane.

**Figure 7:**
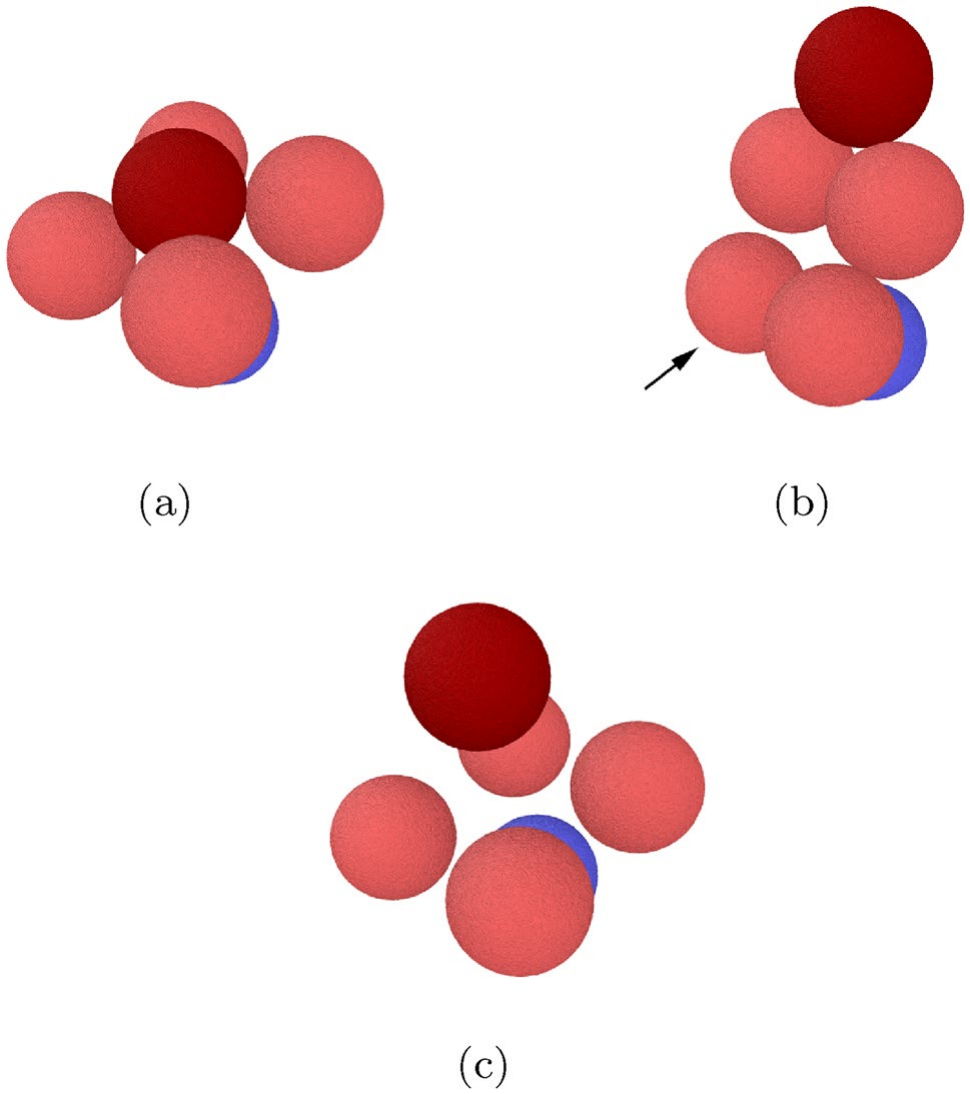
Snapshots showing the collision outcomes for a pentamer collision: (**a**) $v= 0.018$ (bouncing); (**b**) $v= 0.036$ (loss of one monomer); (**c**) $v=0.072$ (destruction). The arrow in subfigure (b) identifies the grain that got detached from the aggregate.

For velocities above the bouncing threshold, the COR, Fig. 5, shows great similarities between the trimer and the pentamer; see the SM for a zoom into the low-velocity region. Above the bouncing velocity, the COR steeply increases, but the maximum value reached is now only around 0.2, considerably smaller than in the case of the monomer. For these collisions with a trimer and a pentamer, the projectile bounces from the intact aggregate. With increasing velocity, the COR decreases to values close to zero; an inspection of the collision dynamics shows that the aggregate is destroyed at these velocities, by pushing out the hit central grain. This process is illustrated in Fig. 6 for the trimer and in Fig. 7 for the pentamer. Note that in the subfigures (a) all bonds in the aggregate are intact; the projectile bounces from the central grain and the aggregate only slightly changes its form from a linear (or planar) structure to a slightly bent shape. In the subfigures (c), however, the projectile is still reflected but the central grain is accelerated away from the aggregate such that all bonds to the neighboring grains are broken. Due to the simple aggregate conformation, this means that the aggregate is totally disrupted to an assembly of isolated grains. We denote by $v_{\textrm{destr}}$ the velocity needed to destroy the aggregate; its values are tabulated in Table 2.

**Figure 8:**
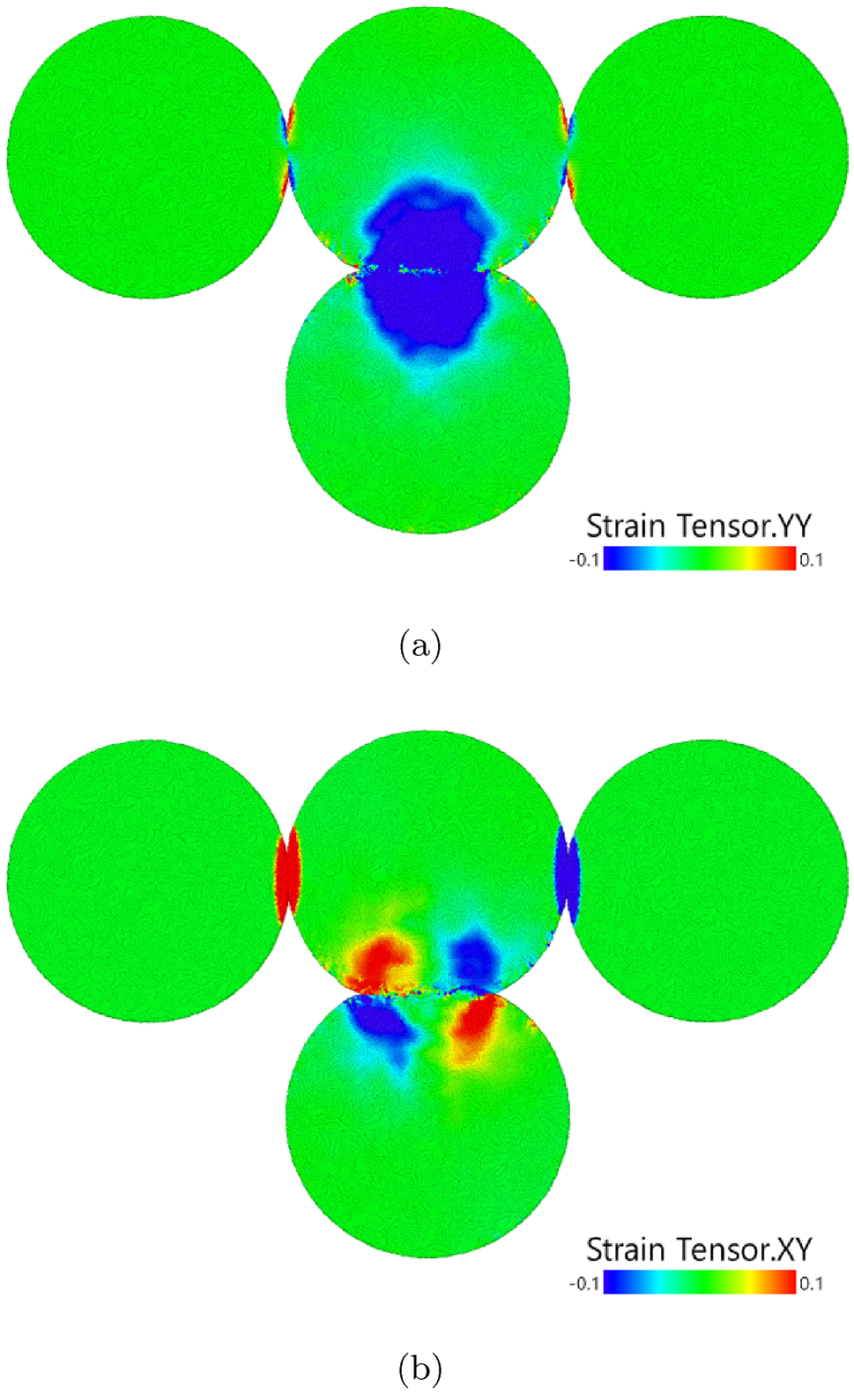
Snapshot of a grain–trimer collision ($v=0.4$) near the time of maximum compression of the projectile grain. Atoms are colored (**a**) with the normal strain in the direction of the collision velocity, (**b**) with the shear strain in the plane formed by the collision velocity and the trimer axis.

For trimer and pentamer aggregates, the destruction velocity is a factor of roughly 5 larger than the bouncing velocity. This large difference may be rationalized by considering in which way the contact between the grains is loaded. Figure 8 shows the strain developing during the collision with the trimer for an exemplary case. A high collision velocity was chosen in order to obtain large strain values and increase the signal-to-noise ratio; however, the qualitative features remain similar for other velocities. Strong compressive pressures build up during the collision in the contact of the projectile with the central grain of the trimer, see Fig. 8a. In the lateral regions of this contact, also shear strains appear due to the sideways atomic motion during compression, see Fig. 8b. In addition shear strain develops in the contacts connecting the central trimer grain with its neighbors. Figure 8 thus illustrates that the contact between projectile and central aggregate – responsible for projectile bouncing – is under both normal and shear load while the contacts between the aggregate grains, which determine aggregate destruction, are exclusively under shear load. These different loading scenarios explain why the threshold velocities for bouncing and destruction assume different values.

For intermediate velocities, an interesting phenomenon – between intact bouncing and total disruption – occurs which may be denoted as ‘monomer loss’^20^, inducing aggregate restructuring. For the pentamer, monomer loss from the aggregate is observed for the velocities of $v=0.036$ and 0.045, while the projectile grain is reflected; Fig. 7b illustrates the restructured aggregate in which the hit central grain has been expelled sideways and now only bonds to 2 other aggregate grains forming a triangular trimer. A fourth constituent grain is bonded to only one of these grains, while the fifth (marked by an arrow) has been expelled and is eventually isolated. The total number of intergranular bonds has remained constant as 4 due to the aggregate restructuring. One may wonder how a central collision leads to the asymmetric final aggregate conformation depicted in Fig. 7b: It is caused by the fact that amorphous grains are not exactly round but possess a surface roughness which makes the aggregate asymmetric. This asymmetry influences the grain dynamics at the small velocities encountered during aggregate restructuring.

Monomer loss is also observed in the trimer. Before the onset of complete destruction, we observe at $v=0.04$ and 0.05 that the trimer partially breaks up into a dimer and a monomer grain, see Fig. 6b.

The onset of aggregate destruction is marked by the pronounced minimum in the COR, Fig. 5. This minimum is understandable from a simple argument based on the idea that the collision of the projectile with the central grain in the aggregate might be described in a first approximation as a ‘spectator collision’ in which the other aggregate grains receive no momentum during the collision. Since both grains have equal mass, the projectile pushes out the central grain while the projectile takes its position in the aggregate, resulting in a replacement collision. This amounts to a velocity transfer of *v*; this ‘shear velocity’ then strains the bonds between the central aggregate grain and its neighbors. This is exactly what we observe at the COR minimum at $v_{\textrm{destr}}$, see Fig. 6. At even higher velocities, the projectile bounces back while the hit central aggregate grain is pushed out. The physics shown in Fig. 6 for the trimer is identical to that in the pentamer in Fig. 7. We show in the Supplementary Material the distribution of strain in the pentamer collision in analogy to Fig. 8; the close analogy in the strain distribution highlights that the physics in the destruction process in trimer and pentamer collisions is similar.

Theoretical considerations of granular aggregate collisions based on granular mechanics argue that the collision energy needed to eject grains from aggregates scales as^17,18,20^

12 $$\begin{aligned} E_{\textrm{destr}}\propto N_c E_{\textrm{sep}}. \end{aligned}$$

This dependence on the number of contacts is not supported by our findings as the destruction energy $E_{\textrm{destr}}$ is only slightly (35 %) higher for the pentamer than for the trimer, even though the number of contacts doubles. We thus conclude that energy dissipation processes in the hit grain may be more decisive for the collision dynamics than bond breaking to neighboring grains.

In more quantitative detail, Dominik and Tielens^20^ predict that an aggregate will lose monomer grains for collision energies $E > \alpha N_c E_{\textrm{sep}}$, where $\alpha$ is a number in the range of 0.3–3, and that the aggregate will disrupt ‘catastrophically’ for $E > 10 N_c E_{\textrm{sep}}$; that means $E_{\textrm{destr}}= 20E_{\textrm{sep}}$ for the trimer ($N_c=2$) and $E_{\textrm{destr}}= 40 E_{\textrm{sep}}$ for the pentamer ($N_c=4$). Our study shows that $E_{\textrm{destr}}= 50E_{\textrm{sep}}$ for the trimer and $E_{\textrm{destr}}= 68 E_{\textrm{sep}}$ for the pentamer, see Table 2, which is at or above the upper end of this estimate.

Wada *et al.*^49,50^ discuss the so-called growth velocity, $v_{\textrm{growth}}$, in the collision of two aggregates. For the collision of a small projectile aggregate with a large target aggregate, it is defined as the smallest velocity which results in a post-collision aggregate that is smaller than the pre-collision target. Thus, we may identify the growth velocity with the destruction velocity in our context. From granular mechanics simulations in a wide range of mass ratios of projectile and target aggregates, Wada *et al.*^50^ find that

13 $$\begin{aligned} v_{\textrm{growth}}= 20 \sqrt{E_{\textrm{sep}}/M} = 10 v_{\textrm{sep}}. \end{aligned}$$

Our simulations find $v_{\textrm{destr}}\cong 7 v_{\textrm{sep}}\cong 5 v_{\textrm{bounce}}$ in not too large disagreement.

**Figure 9:**
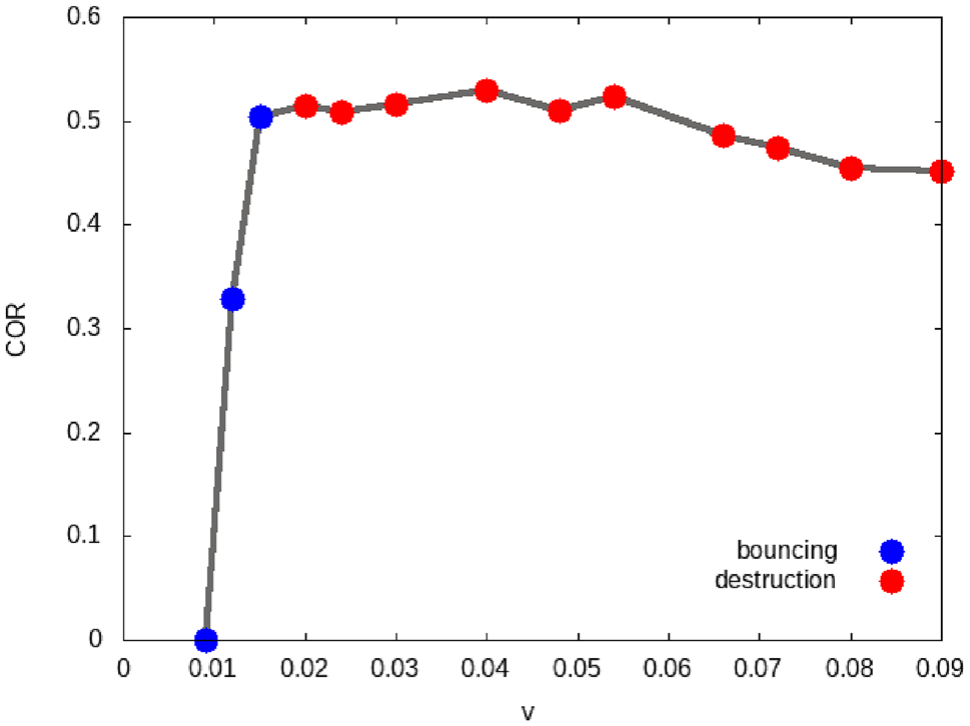
Velocity dependence of the coefficient of restitution (COR) for grain–dimer collisions. Data are colored according to the collision outcome: bouncing from the intact aggregate (blue), destruction (red).

### Grain–dimer collisions

The collision of a grain with a dimer, Fig. 1b, consisting of grains 1 and 2 differs from the collisions discussed previously in that the projectile does not collide centrally with a constituent grain; rather it performs an oblique collision with each of the constituent grains 1 and 2.

Table 1 shows that that the bouncing velocity for the dimer is more or less identical with those of a monomer or the other aggregates discussed above. FigURE 9 shows that the entire shape of the COR dependence on velocity closely resembles that for grain–grain collisions, Fig. 4; only the COR values for the dimer are somewhat reduced compared to the monomers.

At the bouncing velocity, the dimer stays intact. We find that only for $v \ge 20\cdot 10^{-3}$, the dimer is dissociated. Thus, the destruction velocity $v_{\textrm{destr}}$ is roughly a factor of 2 higher than the bouncing velocity. We may understand that from the following argument. Since the projectile grain velocity is directed towards the center of mass of the dimer, it simultaneously hits both dimer grains. At the moment of contact, the three grains form an equilateral triangle. The vector of the relative velocity ${\varvec{v}}$ may be split up into two vectors directed along the projectile-1 and projectile-2 axis, ${\varvec{v}}= {\varvec{v}}_1 + {\varvec{v}}_2$; according to elementary trigonometry, it is $v_1 = v_2 = (\sqrt{3}/4)v = 0.43v$. In a first approximation, we might therefore expect that the collision with the dimer equals the collision with a monomer at reduced velocity 0.43*v* and that the destruction velocity will therefore be enhanced by a factor 1/0.43=2.3. This value is indeed close to the increase of the destruction velocity for monomer to dimer collisions.

## Summary and conclusions

We studied the collision behavior of amorphous grains with small aggregates by atomistic simulations and found the following features.

1. The separation energy $E_{\textrm{sep}}$ needed to break the contact between two grains corresponds well with the predictions of macroscopic continuum theory of contacts.
2. With increasing collision velocity, we observe a transition from sticking to bouncing of the projectile grain. It bounces first from the intact aggregate, then from a restructured aggregate which loses a monomer grain, and at the highest velocities from a completely disrupted aggregate.
3. The bouncing energy $E_{\textrm{bounce}}$ describing the minimum collision energy necessary to let two colliding grains bounce from each other surpasses the separation energy by a factor of around 2. This demonstrates that energy dissipation processes during the collision influence the collision dynamics and exceed the dissipation during breaking a contact.
4. Central collisions of a grain with a granular aggregate feature approximately the same bouncing velocity as grain–grain collisions.
5. At sufficiently high velocities, aggregates are destroyed in the collision by kicking the hit grain out. For trimer and pentamer aggregates, the destruction velocity is a factor of roughly 5 larger than the bouncing velocity.
6. In a narrow velocity window below total aggregate destruction, the aggregate loses a single monomer and restructures by breaking bonds and forming new bonds.
7. The destruction energy $E_{\textrm{destr}}$ is only slightly (35 %) higher for the pentamer than for the trimer. This is in contrast to theoretical predictions which postulate that $E_{\textrm{destr}}$ increases in proportion to the number of contacts that need to be broken; the pentamer should therefore need double the collision energy of the trimer for complete destruction. This feature shows that energy dissipation processes in the hit grain may be more decisive for the collision dynamics than bond breaking to neighboring grains.
8. Central collisions of a grain with a dimer hit the dimer grains obliquely. Nevertheless, the bouncing velocity is the same as that for the monomer or for larger aggregates. However, the destruction energy is strongly decreased (by an order of magnitude) compared to the larger aggregates. This is caused by the fact that the momentum imparted to the dimer grains is partly parallel to the dimer axis and thus assists dimer dissociation.

A important finding is thus that the role of energy dissipation strongly surpasses that of contact breaking. Energy dissipation processes will however be strongly dependent on the material constituting the grains. A generalization of our results to other – more realistic – materials has therefore to be done with caution. From previous atomistic studies it is known that phase transformations may strongly affect the collision process – such as melting in water ice^24^ and $\hbox {sp}^3$-$\hbox {sp}^2$ hybridization (graphitization) in amorphous carbon grains^51^. In crystalline grains, the generation of dislocation plasticity additionally influences the collision dynamics^35,47,52^.

Our results also hint at the different role played by contacts loaded perpendicular to the contact area (as in grain–monomer collisions) and contacts loaded tangentially to the contact area (as in trimer and pentamer collisions). The dimer collisions are intermediate between these two extremes. While the bouncing velocity is mainly dictated by the normally loaded contact of the projectile with the hit target grain – and leads to similar bouncing velocities in all cases studied –, the destruction velocity is governed by the shear loading of the lateral contacts of the hit grain with its neighbors in the aggregate. Their different response leads to the large values of the destruction velocity as compared to the bouncing velocity.

Interestingly, our atomistic results on the evolution of collision outcomes with increasing collision velocity – sticking to the original aggregate, bouncing from the intact aggregate, loss of a single monomer, complete disruption of the aggregate – is the same as that assumed in theoretical considerations and found in granular mechanics simulations^49,50^. The main advantage of our atomistic study is that it allows to determine the collisional energy loss directly, while assumptions about the importance of various contributions – such as the energy dissipation in plastic processes or the excitation of elastic waves – have to be made granular mechanics calculations^45^. Our results show that even in the collision of two monomer grains, the energy loss surpasses the energy needed for grain separation.

The atomistic simulations presented here thus shed new light on the basis of granular mechanics and invite to rethink its postulates. In particular, we mention:

(i) The equivalence of the separation energy and the bouncing energy needs be questioned due to the occurrence of energy dissipation processes during grain bouncing.
(ii) The energy loss of two grains during bouncing is not constant but depends on the collision energy, in contrast to the common assumption in granular mechanics.
(iii) The idea that grain bouncing from aggregates requires the same energy irrespective of aggregate size has been corroborated.
(iv) The energy needed for aggregate disruption is around a magnitude larger than the separation energy, in agreement with granular mechanics results^20,49,50^.
(v) The idea that the destruction energy of an aggregate increases in proportion to the number of bonds to be broken is not necessarily true for small aggregates.

Our results have been obtained for central collisions only, in which the grain impacts on the center of mass of the aggregates, and for small symmetrical aggregates. Due to the expensive computation costs of atomistic simulations, a generalization of the results to non-central and oblique collisions will not be easily done. Also, further investigations on the influence of the granular surface energy $\gamma$ – here modeled by the intergranular attraction $\epsilon _{12}$ – and the grain radius *R* on the results will be welcome.

### Supplementary Information

Supplementary Figures.

## Data Availability

All data used for this study are contained in this article. Pertinent LAMMPS input scripts can be found in https://guriang.unpad.ac.id/refs/aggrcols/.
