# Supplementary material for: An atomistic study of sticking, bouncing, and aggregate destruction in collisions of grains with small aggregates
Source: Sci Rep. 2024 Mar 28;14:7439. doi: 10.1038/s41598-024-57844-y (PMC10978963; doi:10.1038/s41598-024-57844-y)
Supplement: Supplementary file 1 — Supplementary Figures. [file 41598_2024_57844_MOESM1_ESM.pdf]

An atomistic study of sticking, bouncing, and aggregate destruction in collisions of grains with small aggregates
Supplementary Material

Maureen L. Nietiadi,[1] Herbert M. Urbassek,[2,*] and Yudi Rosandi[1]

[1]*Department of Geophysics, Universitas Padjadjaran, Jatinangor, Sumedang 45363, Indonesia*
[2]*Physics Department, University Kaiserslautern-Landau, Erwin-Schrödinger-Straße, D-67663 Kaiserslautern, Germany*

(Dated: February 21, 2024)

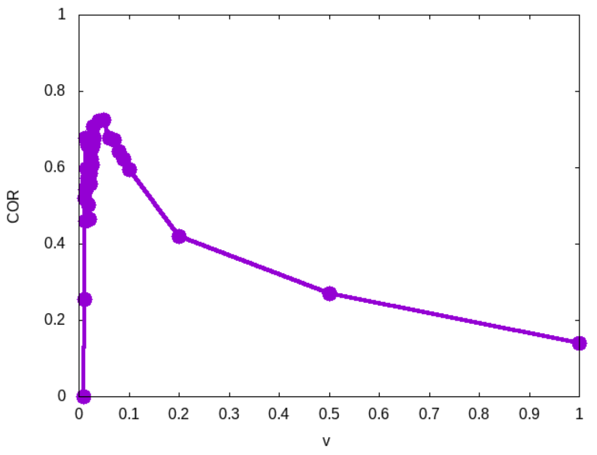

FIG. S1: Velocity dependence of the coefficient of restitution (COR) for grain-grain collisions. Data are over the full velocity range simulated and extend Fig. 3a.

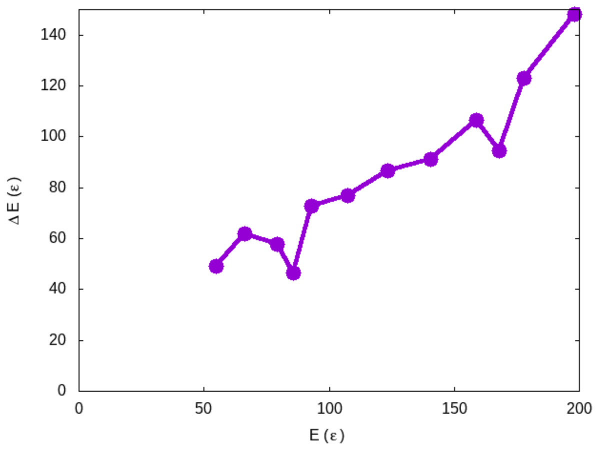

FIG. S2: Zoom into the energy dependence of the energy loss for grain-grain collisions (Fig. 3b) for small collision energies E.

*Electronic address: urbassek@rhrk.uni-kl.de; URL: http://www.physik.uni-kl.de/urbassek/

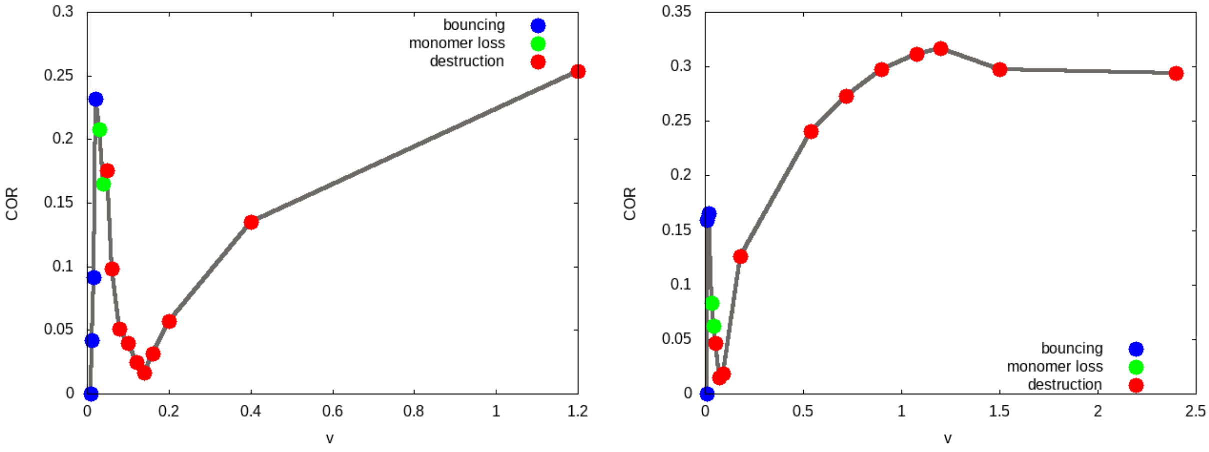

(a) (b)

FIG. S3: Velocity dependence of the coefficient of restitution (COR) for grains colliding centrally with a (a) trimer, (b) pentamer. Data are colored according to the collision outcome: intact reflection (blue), restructuring or partial destruction (green), total destruction (red). Data are over the full velocity range simulated and extend Figs. 4a and b.

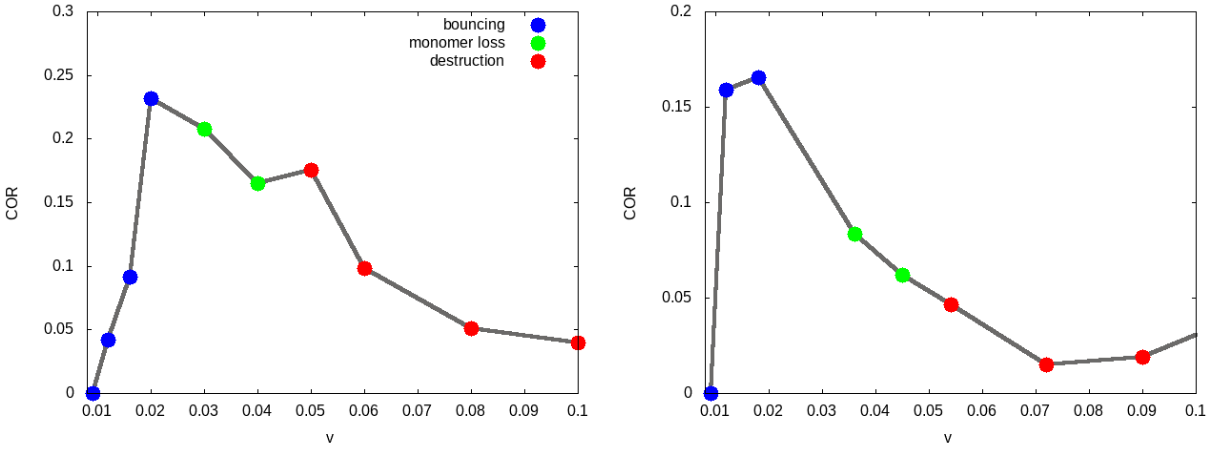

(a) (b)

FIG. S4: Zoom into the low-velocity range of Figs. 5a and b.

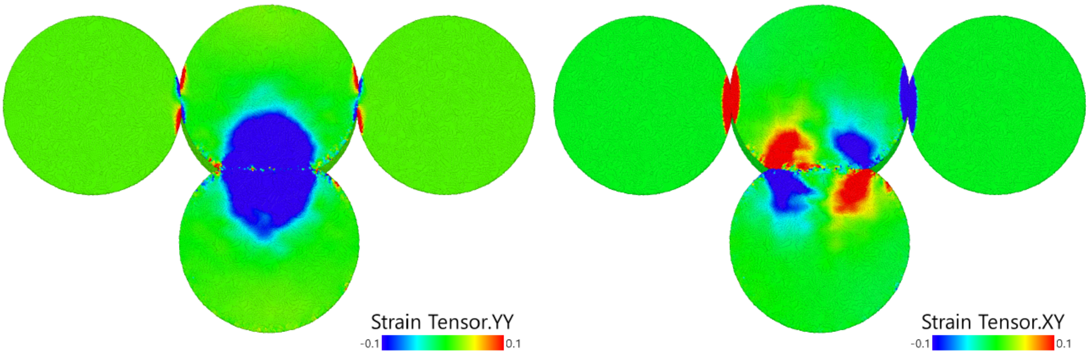

(a) (b)

FIG. S5: Snapshot of a grain-pentamer collision ($v = 0.54$) near the time of maximum compression of the projectile grain. Atoms are colored (a) with the normal strain in the direction of the collision velocity, (b) with the shear strain in the plane formed by the collision velocity and one of the pentamer axes. The strains in a plane cutting through the other pentamer axis are identical by symmetry. This figure is analogous to Fig. 8, but for a pentamer.
